# Gastrointestinal Biopsies for the Diagnosis of Alpha-Synuclein Pathology in Parkinson's Disease

**DOI:** 10.1155/2015/476041

**Published:** 2015-05-11

**Authors:** Maria Graciela Cersosimo

**Affiliations:** Parkinson Disease and Movement Disorders Unit, Hospital de Clínicas, University of Buenos Aires, GCBA, C1120AAR Buenos Aires, Argentina

## Abstract

The diagnosis of Parkinson's disease (PD) relies on clinical features whereas pathological confirmation is only possible with autopsy examination. The neuropathological hallmarks of PD are neuronal loss and the presence of inclusions termed Lewy bodies/neurites in affected regions. A major component of these inclusions is phosphorylated alpha-synuclein (*α*-SYN) protein. There is evidence that *α*-SYN pathology is widely distributed outside the central nervous system in patients with PD. The gastrointestinal tract is importantly affected by *α*-SYN containing inclusions and typically there is a rostrocaudal gradient for the distribution of the pathology. The highest amounts of Lewy bodies/neurites are found at the submandibular gland together with the lower esophagus and the lowest amounts are found in the rectum. Autopsy findings prompted research aimed at achieving in vivo pathological diagnosis of PD by demonstrating the presence of *α*-SYN pathology in biopsy material of these peripheral accessible tissues. So far, biopsy studies of the gut have demonstrated the presence of *α*-SYN pathology in the salivary glands, stomach, duodenum, colon, and rectum. Further research is necessary in order to determine which are the most sensitive targets for in vivo *α*-SYN pathology detection and the safest techniques for these approaches in patients with PD.

## 1. Background

Parkinson's disease (PD) is one of the most common neurodegenerative diseases in the elderly and the most frequent of synucleinopathies. It is clinically characterized by the presence of motor as well as nonmotor manifestations. Typical motor symptoms of PD are bradykinesia, resting tremor, rigidity, and late postural instability [1]. In addition, a wide variety of nonmotor manifestations, such as autonomic, sleep, cognitive, and olfactory dysfunctions, are part of the clinical spectrum of the disease [2]. Among autonomic problems, gastrointestinal manifestations are prominent and disabling symptoms of PD and their presence has been recognized since the early descriptions of the disease [3, 4].

The central nervous system (CNS) pathological hallmark of PD is the loss of dopaminergic neurons in the substantia nigra pars compacta and the presence of Lewy pathology (LP) in affected brain regions [5]. LP comprises cytoplasmic spherical eosinophilic inclusions in the perikarya of nerve cells termed Lewy bodies and spindle or thread-like inclusions known as Lewy neurites, localized within neuronal processes [5]. A major component of LP is abnormal phosphorylated alpha-synuclein (*α*-SYN) protein [6, 7].

To date, the diagnosis of PD relies mostly on clinical features whereas neuropathological confirmation is only possible with postmortem studies. In the last years, numerous studies demonstrated that *α*-SYN pathology in PD is widely distributed throughout the body [8, 9]. The presence of LP outside the CNS was described in the submandibular gland, the enteric nervous system, the sympathetic ganglia, the cardiac and pelvic plexuses, the adrenal medulla, and the skin [8–11]. These findings led to research aimed at achieving pathological confirmation of PD during life by biopsying these accessible peripheral tissues, particularly the submandibular gland and the enteric nervous system [12–15]. This is of interest as it raises the possibility of in vivo demonstration of *α*-SYN histopathology, therefore improving the accuracy of PD diagnosis.

This paper aims to briefly review (i) the place of PD in the setting of synucleinopathies; (ii) the lines of evidence of gastrointestinal tract involvement by *α*-SYN pathology in autopsy studies; and (iii) the feasibility of gastrointestinal biopsies for the detection of *α*-SYN pathology in PD living patients.

## 2. The Synucleinopathies Spectrum

*α*-SYN is a natively unfolded soluble 140-amino-acid protein highly expressed in the CNS. Although the precise physiological function of *α*-SYN remains unclear, there are multiple lines of evidence suggesting a role in synaptic vesicle trafficking [16]. The term* synucleinopathies* refers to a group of neurodegenerative diseases characterized by the presence of abnormal misfolded *α*-SYN containing inclusions in the CNS; PD is the most common of this group of diseases. Classically, two major groups of synucleinopathies are described: LP disorders and multiple-system atrophy (MSA). LP disorders are defined by the presence of intraneuronal Lewy bodies/neurites; this group includes PD, dementia with Lewy bodies (DLB), and pure autonomic failure [16]. In MSA, *α*-SYN inclusions are mainly, but not exclusively, found in the cytoplasm of oligodendroglial cells. Both LP disorders and MSA are associated with gastrointestinal dysfunction; however, the compromise of the autonomic nervous system in LP disorders is primarily postganglionic whereas in MSA it is preganglionic [17].

## 3. Autopsy Studies: ENS Involvement by *α*-SYN Pathology in PD

Qualman et al. described for the first time the presence of Lewy bodies in the enteric nervous system (ENS) of PD cases; the authors identified inclusions in the esophagus of one PD case and the colon of another [18]. Since then, numerous pathological studies of PD were published showing that LP is widely distributed in Auerbach's and Meissner's plexuses involving almost the entire gut [19–21].

There is a rostrocaudal gradient of LP within the gastrointestinal system with the highest amount of pathological aggregates in the upper compared to the lower gut. Wakabayashi et al. first observed this distribution of LP in a study of seven PD cases using classical staining techniques [19]. More recently, Beach and coworkers [8] employing a sensitive immunohistochemical method for the detection of phosphorylated *α*-SYN pathology confirmed the rostrocaudal gradient of *α*-SYN histopathology within the gastrointestinal tract [8]. In this study the submandibular gland and the lower esophagus showed the greatest involvement by *α*-SYN aggregates, followed by the stomach, small bowel, colon, and rectum [8]. Consistent with this study, Del Tredici et al. confirmed that LP importantly affects the submandibular gland; in these studies LP was present in 9 out of 9 and 22 out of 23 PD cases, respectively. In addition, Del Tredici et al. found *α*-SYN pathology in the submandibular gland of incidental LP cases, suggesting that its compromise might occur early in the course of the disease [11].

It has been suggested that the rostrocaudal gradient of *α*-SYN pathology along the ENS is probably related to vagal innervation of the gut. The vagus innervates the gastrointestinal tract as far as the proximal colon; however, its major influence is exerted in the lower esophagus and stomach whereas small bowel and colon are mostly under the influence of the ENS [22–24]. An exception to the rostrocaudal gradient of LP is the upper third of the esophagus, which was found to be free of *α*-SYN inclusions in all cases examined [8]. A possible explanation for this could be that vagal axons innervating the upper esophagus originate in the nucleus ambiguously, which is not affected by *α*-SYN pathology [8].

In addition to the* topographic related vulnerability* described for LP along the ENS,* neuron type vulnerability* has been also shown. Most Lewy neurites in the gastrointestinal tract are localized in the processes of vasoactive intestinal polypeptide (VIP) immunoreactive neurons [20]. The reason for VIP neurons susceptibility within the ENS of PD cases is poorly understood. Interestingly, whereas dopaminergic neurons in the myenteric plexus are markedly reduced in cases with advanced PD, the number of VIP neurons is not significantly decreased [10].

### 3.1. The Braak Hypothesis: Possible Route of *α*-SYN Pathology from the Gut to the CNS

Braak et al. formulated the hypothesis that an exogenous pathogen might gain entry through the gastrointestinal tract, trigger abnormal *α*-SYN processing, and spread retrogradely from ENS to the CNS via axons of the dorsal motor nucleus of the vagus (DMV) [25]. In the brain the neuropathological process would progress in six stages with the earliest lesions appearing at the DMV and the olfactory bulb. From the DMV *α*-SYN aggregates would spread in a predictable and stereotype manner progressing to the midbrain and finally to the basal forebrain and neocortex in a caudorostral progression [26–28]. Interestingly *α*-SYN pathology would progress affecting selectively vulnerable type of neurons. Vulnerability appears to be related to axonal length and myelination. In PD, cells predisposed to develop *α*-SYN pathology are those with long and thin axons, together with poor or incomplete myelination [25]. At stage 3 of Braak's model occurs the compromise of the substantia nigra pars compacta together with the amygdala and the magnocellular nuclei of the basal forebrain; as a result motor symptoms and clinical diagnosis of PD occur at this stage. At Braak's stages 1 and 2 *α*-SYN pathology is found in the lower brainstem and the DMV; these cases comprise the so-called* incidental *LP cases which likely represent early premotor PD cases [26–28]. Moreover, according to Braak et al., incidental cases already present *α*-SYN pathology at the ENS, suggesting that the disease starts outside the CNS and before stage 1 of the model [25]. Together with the ENS, incidental cases also present abundant LP in the submandibular gland [11].

This hypothesis has gained growing interest as it provides the basis for the understanding of gastrointestinal symptoms as premotor manifestations of PD. In addition it raises the possibility of making early pathological diagnosis of PD by means of biopsying these peripheral tissues like the submandibular gland or the gastrointestinal tract.

The Braak staging for the neuropathology in PD is supported by other pathological studies [29, 30], whereas others failed to reproduce the same results [31]. Whether ENS compromise occurs before or together with the CNS involvement is still controversial.

### 3.2. Misfolded *α*-SYN: A Prion-Like Protein?

There is a growing body of evidence indicating that misfolded *α*-SYN is a prion-like protein and therefore PD could be a prion-like disorder. The Braak model suggests that an exogenous pathogen might trigger abnormal *α*-SYN processing in the gastrointestinal tract and spread from the gut to the CNS via axons of the DMV passing from neuron to neuron in a predictable manner [25].

It has been demonstrated that embryonic tissue grafted into the brain of PD patients showed the presence of *α*-SYN pathology more than ten years later in postmortem examination, suggesting that *α*-SYN aggregates are capable of spreading from host to donor neurons [32, 33]. In a recent study, the inoculation of *α*-SYN pathology obtained from substantia nigra of postmortem PD cases into the brain of unaffected animals was followed by spread of the abnormal inclusions to distant brain regions [34]. Another study showed that the injection of *α*-SYN aggregates in the striatum of mice led not only to spreading of the pathology but also to neuronal loss and motor impairment [35]. These findings led to the increasingly accepted idea that misfolded *α*-SYN behaves in a prion-like manner. Similar to what is observed in prion diseases, in PD, misfolded *α*-SYN triggers the conversion of normal endogenous *α*-SYN into its abnormal beta-sheet conformation initiating a progressive neurodegenerative process. However, whereas the propagation mechanisms are similar for both prions and prion-like proteins, only prions are infectious [36, 37]. Nevertheless, this is an ongoing line of research and the topic is not completely elucidated; thus proceeding with caution would be prudent when invasive procedures are undertaken among PD patients [38].

## 4. In Vivo Studies: Biopsies for *α*-SYN Pathology Detection in the Gut of PD Patients

### 4.1. Biopsy Studies

Several recent studies suggest that biopsies of the gastrointestinal tract and the submandibular gland constitute a promising approach to detect *α*-SYN neuropathology in autonomic nerves of PD living cases [12–15, 39, 40]. In addition, the compromise of the ENS and the submandibular gland by *α*-SYN pathology is likely to occur very early in the course of the disease [11, 25]; thus biopsies of these tissues could potentially be useful as early biomarkers of PD. So far, abnormal *α*-SYN accumulations have been identified in biopsies of the salivary glands [13, 15, 41], stomach, duodenum [40, 42], colon, and rectum of PD patients [12, 14, 39, 43].

#### 4.1.1. Salivary Glands

Salivary glands provide an attractive target for biopsies as the highest amount of *α*-SYN aggregates in autopsy studies of PD cases was found in the submandibular gland [8, 11].

Cersosimo et al. conducted a pilot study of biopsies of the minor salivary glands showing *α*-SYN inclusions in 2 out of 3 PD patients and none of 3 controls [13]. In this study labial minor salivary gland biopsy was performed according to the standard procedure used for diagnosis of Sjögren disease, which represents a relatively simple and safe approach. Two further studies were conducted in order to assess the feasibility of minor salivary glands biopsies for *α*-SYN pathology detection.

Folgoas et al. in a larger study performed biopsies of the minor salivary glands in 16 PD patients but found the presence of LP in only 3 of them (18.7%); therefore, it was concluded that despite being technically simple, biopsies of the minor salivary glands are not sensitive for LP detection in patients with PD [41].

Another study conducted by Adler and coworkers assessed the presence of *α*-SYN pathology in biopsies of both the submandibular gland and the minor salivary glands in 15 PD patients [15]. Submandibular biopsy was performed unilaterally under local anesthesia. The gland was localized by palpation and a biopsy needle was inserted transcutaneously. The authors found LP in 9 out of 12 (75%) patients in the submandibular gland and in 1 out of 15 (6.7%) in the minor salivary glands. In 3 cases, the material obtained by needle biopsy of the submandibular gland was devoid of gland tissue. This study demonstrated that the submandibular gland biopsy is a sensitive tool for diagnosis of LP in PD patients. On the contrary, but according to previous results [41], sensitivity of minor salivary glands was low [15].

#### 4.1.2. Stomach and Duodenum

Pouclet et al. performed gastrointestinal biopsies in one PD patient undergoing an endoscopic procedure during device implantation for continuous levodopa enteral infusion. Lewy neurites immunoreactive for phosphorylated alpha-synuclein were found in fundic, antral, and duodenal submucosa [42].

In a recent study, Sánchez-Ferro et al. assessed the presence of *α*-SYN inclusions in biopsies of gastric mucosa of 28 PD patients, 23 controls, and 6 cases with premotor symptoms [40]. All PD cases presented motor fluctuations and gastric biopsy samples were obtained during percutaneous endoscopic gastroscopy in order to initiate continuous levodopa enteral infusion therapy. Mucosal biopsies were obtained from the antral and pyloric regions. In 8 PD cases the gastric mucosa specimens had inadequate quality to be processed for neuropathological study. The authors found LP in 17 out of 28 PD cases biopsied (60.7%); 1 of 23 controls (4.3%); and 1 of 6 (16.7%) presymptomatic cases. The pyloric region was the richest in *α*-SYN aggregates. Interestingly this study was aimed at performing superficial biopsies of gastric mucosa, instead of submucosa, in order to minimize possible risks [40].

#### 4.1.3. Colon and Rectum

Lebouvier et al. published the first study of gut biopsies in PD living patients. The authors demonstrated for the first time the presence of Lewy neurites in the submucosal plexus of biopsies obtained from the ascending colon of 4 out of 5 PD patients. LP was absent in 5 healthy controls and in 3 controls with chronic constipation [12].

In a larger study, the same group performed biopsies of the ascending and descending colonic submucosal plexus in 29 PD patients and 10 control subjects. Immunohistochemical staining for phosphorylated *α*-SYN demonstrated LP in the submucosal plexus in 21 out of 29 (72%) PD cases whereas in controls LP was absent [39]. The burden of *α*-SYN inclusions in PD cases showed a positive correlation with age, disease duration, Unified Parkinson's Disease Rating Scale (UPDRS) score, and the presence of levodopa unresponsive features and constipation [39]. The authors published a further study based on the same patients' colonic biopsies samples aimed at comparing the sensitivity between ascending colon, descending colon, and rectum [44]. The results showed that the ascending colon presented LP in 17 out of 36 (65%) patients, the descending colon in 11 out of 26 (42%), and the rectum in 6 out of 26 (23%). These results are consistent with autopsy studies observations demonstrating a rostrocaudal distribution of LP within the gastrointestinal tract; in addition, they show the relatively low sensitivity of the rectum for LP detection in PD patients [44]. In another study, the authors compared the feasibility of colonic mucosa versus submucosa for the detection of LP among 10 PD patients who underwent rectosigmoidoscopy [45]. Lewy neurites were observed in the submucosal plexus of 4 PD patients compared to 3 cases with LP in the mucosa. Interestingly, one PD patient who exhibited Lewy neurites in the colonic mucosa was devoid of LP in the submucosa suggesting that the analysis of both mucosa and submucosa can improve the chance of detecting *α*-SYN pathology [45].

Shannon et al. assessed biopsies obtained from sigmoid colon submucosa in 10 PD patients, 23 healthy controls, and 23 patients with inflammatory bowel disease [14]. Importantly, for rectosigmoidoscopy neither colon preparation nor anesthesia is necessary. In this study, the antibody used for immunohistochemical staining was not specific for phosphorylated *α*-SYN. The results showed that 9 out of 10 PD cases presented a pattern strongly consistent with the presence of nerve fibers, which was not observed in any of the control cases (in one PD case the biopsy material was insufficient) [14]. The authors conducted another study, analyzing distal colonic biopsies from 3 PD cases that had been performed 2 to 5 years before the onset of PD motor symptoms because of clinical reasons; the same 23 healthy controls from the previous study were included. Similar to the previous study, the antibody employed was not specific for phosphorylated *α*-SYN. All PD patients, but not controls, showed intense *α*-SYN immunostaining in colonic biopsies. This is the first study demonstrating *α*-SYN pathology in the gut of PD patients before the onset of motor symptoms [43].

### 4.2. Surgical Specimens

In vivo demonstration of *α*-SYN pathology outside the CNS in PD patients is also possible by analyzing surgical specimens obtained during interventions for diverse pathological conditions. Minguez-Castellanos et al. detected LP in 6 out of 100 subjects without clinical manifestations of any neurological disease, who underwent abdominopelvic resections [46]. These cases as well as a control group were followed up. Thirty months after surgery, the group of patients that presented *α*-SYN pathology in surgical specimens showed a significant reduction in cardiac [(123)I] metaiodobenzylguanidine uptake and in striatal [(123)I] ioflupane uptake. In addition UPDRS motor score was significantly higher in cases with LP compared to controls [46]. These findings strongly indicate that *α*-SYN pathology detected in peripheral tissues of these cases preceded autonomic dysfunction and motor manifestations associated with the onset of Lewy body disease.

A recent study demonstrated the presence of LP in gastrointestinal and biliary surgical specimens in 6 out of 8 (75%) patients with diagnosis of Lewy body disease, either PD with dementia or dementia with Lewy bodies [47]. In two of these cases, surgical interventions had been performed 2 and 7 years before the onset of neurological symptoms. The authors emphasized the potential utility of surgical specimens obtained during gastrointestinal or biliary interventions, for the confirmation of *α*-SYN pathology in patients with clinical features of Lewy body disease, regardless of whether neurological symptoms began before, together with, or even after the surgical procedure [47].

## 5. Perspectives and Conclusions

The compromise of the gastrointestinal tract by LP in PD has been a matter of increasing interest during the last decade. The Braak hypothesis on the pathodynamics of *α*-SYN pathology, spreading from the ENS to the DMV, has prompted research aimed at elucidating issues related to early diagnosis, pathogenic mechanisms, and pathological confirmation of PD. Biopsies of the submandibular gland and the ENS appear to be a promising tool for in vivo demonstration of *α*-SYN pathology in PD patients and will likely improve diagnostic accuracy. A large number of cases need to be studied in order to learn the actual sensitivity and specificity of these procedures. Biopsies are minimal invasive techniques and, therefore, not entirely devoid of risks; in consequence, the answer to all these uncertainties will probably come in the long term. Further studies are necessary to determine which are the most sensitive targets for *α*-SYN pathology detection and which are the safest techniques for these approaches.
